# Evaluation of the immunogenicity of liposome encapsulated HVR1 and NS3 regions of genotype 3 HCV, either singly or in combination

**DOI:** 10.1186/1743-422X-9-74

**Published:** 2012-03-27

**Authors:** Gouri M Gupte, Vidya A Arankalle

**Affiliations:** 1 Hepatitis Division, National Institute of Virology, Microbial Containment Complex, Sus Road, Pashan, Pune, India 411021; 2 Hepatitis Group Leader, National Institute of Virology, Microbial Containment Complex, Sus Road, Pashan, Pune, India 411021

**Keywords:** Hepatitis C virus, HVR1, NS3, Vaccine, Liposomes

## Abstract

**Background:**

Hepatitis C virus displays a high rate of mutation and exists as a quasispecies in infected patients. In the absence of an effective universal vaccine, genotype-specific vaccine development represents an alternative. We have attempted to develop a genotype 3 based, liposome encapsulated HCV vaccine with hypervariable region-1 (HVR1) and non-structural region-3 (NS3) components.

**Results:**

HCV RNA extracted from serum samples of 49 chronically infected patients was PCR amplified to obtain HVR1 region. These amplified products were cloned to obtain 20 clones per sample in order to identify the quasispecies pattern. The HVR1 consensus sequence, along with three variants was reverse transcribed to obtain peptides. The peptides were checked for immunoreactivity individually, as a pool or as a single peptide tetramer interspersed with four glycine residues. Anti-HCV positivity varied from 42.6% (tetramer) to 92.2% (variant-4) when 115 anti-HCV positive sera representing genotypes 1, 3, 4 and 6 were screened. All the 95 anti-HCV negatives were scored negative by all antigens. Mice were immunized with different liposome encapsulated or Al(OH)_3 _adjuvanted formulations of HVR1 variants and recombinant NS3 protein, and monitored for anti-HVR1 and anti-NS3 antibody titres, IgG isotypes and antigen specific cytokine levels. A balanced Th1/Th2 isotyping response with high antibody titres was observed in most of the liposome encapsulated antigen groups. The effect of liposomes and aluminium hydroxide on the expression of immune response genes was studied using Taqman Low Density Array. Both Th1 (IFN-gamma, Il18) and Th2 (Il4) genes were up regulated in the liposome encapsulated HVR1 variant pool-NS3 combination group. In-vitro binding of the virus to anti-HVR1 antibodies was demonstrated.

**Conclusion:**

The optimum immunogen was identified to be combination of peptides of HVR1 consensus sequence and its variants along with pNS3 encapsulated in liposomes, which could generate both cellular and humoral immune responses in mice deserving further evaluation in a suitable cell culture system/non-human primate model.

## Introduction

Hepatitis C virus (HCV), a major causative agent of chronic hepatitis is distributed worldwide with an estimated 170 million carriers. HCV displays a high rate of mutation contributed by both host and viral components [1-4] and exists in infected patients as a quasispecies, which fluctuate during the course of infection. The extreme sequence variability among different genotypes and the global genotype distribution pattern [5] are major concerns in vaccine development. The observed absence of protection of chimpanzees against reinfection with homologous or heterologous strains is noteworthy [6]. Though the development of an effective vaccine for HCV has been a challenging task, several attempts have been made, a few reaching different clinical trial phases [7]. In the absence of an effective universal vaccine, genotype-specific vaccine development represents an alternate strategy. In India, an estimated 12 to 13 million people are HCV infected [8] with a predominance of genotype 3 (> 70%) [[9], our unpublished data].

The 27 amino acid long N-terminal region of E2 glycoprotein (amino acids 384-410) termed as hypervariable region 1 (HVR1), is the most variable region of the whole HCV polyprotein and contains a neutralizing determinant [10-13]. Studies have shown that HVR1 can serve as B and T cell epitopes and genetic hypervariability is driven by host's immune pressure. Various studies have proven the direct role of anti-HVR1 antibodies in viral clearance [14-17]. Therefore, we evaluated the HVR1 region for candidate vaccine development. However, immune response to envelope proteins develops slowly and achieves only modest titres during primary infection and use of an additional HCV protein was considered necessary [18,19]. In this context, non-structural protein-3 (NS3) eliciting strong humoral and cellular immune responses was chosen. Importantly, the T-helper immune response against NS3 is associated with viral clearance and absence of such response leads to viral persistence [20,21]. Hence, NS3 may be effectively used for vaccine development. This study reports our attempts to develop a genotype-3-based HVR1 and NS3 combination vaccine.

## Materials and methods

### HVR1 peptide and DNA preparations

#### Clinical specimens

Serum samples of 49 HCV RNA positive chronic hepatitis C patients genotyped earlier and stored at -80°C were used.

### RNA extraction, PCR amplification, sequencing and cloning

Following RNA extraction (QIAamp viral RNA method, Qiagen Hilden, Germany) and cDNA generation, HVR1 (1410 to 1610 nt) was amplified using following primers: SS5 (5'-GGGATATGATGATGAAYTGGT-3', sense), SS6 (5'-TCTRGGTGSRTAGTGCCAGCA-3', antisense) for first PCR, and SS7 (5'-TCCATGCARGGCAAYTTGGGG-3', sense), SS8 (5'-GGCAGTRCTGTTRATGTGCCA-3', antisense) for nested PCR. The 200 bp amplicon was sequenced using Big Dye Terminator cycle sequencing technology and ABI3130xl genetic analyzer (Applied Biosystems California, USA). For identification of quasispecies, all PCR amplified products were TA cloned using pGEMT EASY vector system (Promega, WI, USA) according to manufacturer's instructions. On an average, 20 clones were sequenced for every sample. The HVR1 sequences translated to obtain amino acid sequences using MEGA 3.1 [22], were aligned and the consensus amino acid at every position of HVR1 region was determined.

### HVR1 Peptide/DNA synthesis

Four individual peptide variants were commercially synthesized (Bioconcept, Gurgaon, India). The corresponding nucleotide sequences of the peptides were synthesized (Bioresource), amplified and cloned in the pVAX1 vector (Invitrogen, Life Technologies, Carlsbad). A single peptide tetramer interspersed with four glycine residues was obtained from Chromus Biotech, (Bangalore, India). The consensus sequence (designated as variant 1, STYTTGGAAAHTASGLTSLFSPGPKQN) along with three other variants of the consensus that differed from the consensus by 5 amino acids (positions 3,8,9,10,11, Variant 2, STTTTGGSVGRTASGLTSLFSPGPKQN), 3 amino acids (positions 3,8,9, variant 3, STHTTGGTVAHTASGLTSLFSPGPKQN) and one amino acid (position 9, variant 4, STYTTGGAVAHTASGLTSLFSPGPKQN) were used further ELISA and mice experiments.

### ELISA for the evaluation of reactivity of HVR1 peptides with human sera samples

ELISA was performed according to the protocol described earlier [23,24] with the following modifications. Maxisorp Nunc ELISA plates were coated with 1 μg/well peptides and incubated for 37°C for 1 hour followed by overnight incubation at 4°C. Anti-HCV positive and anti-HCV negative human serum samples were added at a dilution of 1:50. A total of 115 anti-HCV positive and 75 anti-HCV negative human sera were tested. The genotype distribution of anti-HCV positive human sera is described in Table 1.

**Table 1:** Performance of HVR1 variants in detecting anti-HCV antibodies

Coating Ag	No positive/no tested				
	**Genotype 3**	**Genotype 1**	**Genotype 4**	**Genotype 6**	**Total**

Variant 1	68/85 (80%)	18/25 (72%)	4/4	1/1	91/115 (79.1%)

Variant 2	61/85 (71.7%)	15/25 (60%)	1/4	0/1	77/115 (67%)

Variant 3	74/85 (87%)	24/25 (96%)	2/4	1/1	101/115 (87.8%)

Variant 4	79/85 (92.9%)	22/25 (88%)	4/4	1/1	106/115 (92.2%)

Pool	63/85 (74.1%)	17/25 68%)	2/4	1/1	83/115 (72.2%)

Tetramer	38/85 (44.7%)	10/25 (40%)	0/4	1/1	49/115 (42.6%)

### Recombinant NS3 protein/DNA

NS3 (1932 bp) of HCV genotype 3a was amplified using primers: NS3F1 (5'-ACCTATACGACCACCTAGCGCCAA-3'), NS3R1 (5'-CACAATCACAACGCAGCCGAC-3') for first PCR, and NS3F2 (5'-GCTTGCGGAGATATTCTTTGCGGGCT-3') and NS3R2 (5'-CCAAGCAACACCCAGGTGCTG-3) for nested PCR and cloned in pVAX1 and pET15b vectors (Invitrogen, Life technologies, Carlsbad). The expressed NS3 protein was purified using Probond purification resin (Invitrogen Life Technologies, Carlsbad) and gel filtration using HPLC (GE Healthcare). The immunoreactivity of NS3 protein was confirmed by western blot using known anti-HCV positive (genotypes 3a, 1b) and anti-HCV negative human sera.

### Immunogen formulations

Table 2 lists immunogen formulations and the doses used. The DNA (nucleotides cloned in pVAX vector) and corresponding protein/peptides (either NS3 and/or HVR1-variant peptides) were encapsulated into liposomes according to the protocol described earlier [23]. The liposomes (cadB) were kindly provided by Cadila Pharmaceuticals (Ahmedabad, India). The antigen formulations were adjuvanted with Al(OH)_3 _as described earlier [25].

**Table 2:** Immunogen formulation and doses

Immunogen	Dosage	Adjuvant used
	25 μg peptide + 5 μg corresponding DNA	cadB
	
HVR1 variant1	50 μg peptide + 5 μg corresponding DNA	cadB
	
	50 μg peptide + 1 μg pNS3	

	25 μg peptide + 5 μg corresponding DNA	cadB
	
HVR1 variant2	50 μg peptide + 5 μg corresponding DNA	cadB
	
	50 μg peptide + 1 μg pNS3	cadB

	25 μg peptide + 5 μg corresponding DNA	
	
HVR1 variant3	50 μg peptide + 5 μg corresponding DNA	
	
	50 μg peptide + 1 μg pNS3	cadB

	25 μg peptide + 5 μg corresponding DNA	cadB
	
HVR1 variant4	50 μg peptide + 5 μg corresponding DNA	cadB
	
	50 μg peptide + 1 μg pNS3	

	Peptide pool + 5 μg DNA corresponding to each peptide	cadB

	Peptide pool + 1 μg pNS3	cadB
	
HVR1 variant pool (25 μg each peptide)	pool + 1 μg pNS3	Aluminium hydroxide
	
	pool + 1 μg pNS3	no adjuvant
	
	Peptide Pool	cadB
	
	Peptide Pool	Aluminium hydroxide
	
	Peptide Pool	no adjuvant

HVR1 variant tetramer	Tetramer 10 μg	aluminium hydroxide
	
	Tetramer 10 μg	cadB

HVR variant tetramer + pNS3	10 μg tetramer + 1 μg pNS3	aluminium hydroxide
	
	10 μg tetramer + 1 μg pNS3	cadB

	1 μg pNS3	cadB
	
	5 μg pNS3	cadB
	
NS3	1 μg pNS3 + 5 μg corresponding DNA	cadB
	
	1 μg pNS3	no adjuvant

### Mice immunizations

This study was approved by the Institutional Ethical Committee for animals and experiments were conducted as per the guidelines. Six- to eight-week-old female Balb/c mice (8 mice/group) were immunized intramuscularly (100 μl) at 0, 4, and 8 weeks interval with various antigens/combinations and different adjuvants (Table 2). The immunogens included the four HVR1 variants given individually, or as a pool of all four variants or as a tetramer. Similarly, recombinant NS3 was used in different combinations. Mice were bled before immunization, and at regular intervals of 2 weeks after first dose and sera were stored at -20°C till tested.

### ELISA for antibodies and isotyping

Recombinant protein based ELISA was performed for detection and titration of anti-NS3 antibodies as described earlier with 1 μg/well pNS3 as the coating antigen [23,24]. For anti-HVR1 antibodies, a similar protocol was used with the following modifications. Coating was done at 37°C for 1 hour followed by overnight incubation at 4°C. Blocking and incubation with mice sera were done at 37°C for 1 hour. Isotyping was done as described previously by using the respective coating antigens [23].

### Cytokine assay

Cytokine assay was performed as described previously [26]. Cytokines in culture supernatants were measured using BD Cytometric Bead Array Mouse Th1/Th2 cytokine kit (BD Biosciences, USA).

### Taqman low density array

Total RNA extraction and gene expression analysis was done from frozen spleen samples as described previously [27]. TLDA card of the mouse immune panel (Applied Biosystems, UK) was used and run on 7900 HT Fast Real-time PCR system (Applied Biosystems, UK) Relative gene expression values were calculated employing comparative Ct method using Applied Biosystems relative quantification (RQ) manager software v1.2. cDNAs from three phosphate buffered saline immunized mice were considered as calibrators. Hprt1 gene was used as endogenous control. Relative quantification values for each study group were used to calculate mean RQ values. For cluster analysis, relative quantification values were log2 transformed and hierarchically clustered with analysis software (cluster 3.0) [28].

### Immune capture RT PCR assay for anti-HVR1 antibodies

The assay was performed as described earlier [29]. RNA from the captured virus was extracted using Qiagen viral RNA kit (Qiagen, Hilden, Germany) and diagnostic PCR was performed using 5'NCR [30].

### Statistical analysis

Student-t test and Mann Whitney tests were performed. SPSS 11.0 software was used for statistical analyses.

## Results

Of the 49 genotype-3 patients' samples used to generate HVR1 consensus sequence, the subtype distribution was 3a (27, 55.1%), 3b (8, 16.3%), 3e (2, 4.1%), 3f (2, 4.1%), 3 g (8, 16.3%), 3i (2, 4.1%).

### HVR1 consensus sequence

A minimum of 1 and a maximum of 12 quasispecies were detected. Amino acid positions 2, 6, 7, 10, 19, 20, 23, and 26 were more conserved while 11, 12, 14, 22 and 27 were most variable. The consensus sequence, variant-1, along with three other variants were evaluated for immunoreactivity and immunogenicity.

Table 1 compares performance of HVR1 variants in detecting anti-HCV antibodies in 115 previously identified anti-HCV positive sera. The percent reactivity for various HVR1-variants was: 79.1% (Variant-1), 67.0% (variant-2), 87.8% (variant-3), 92.2% (variant-4), 72.2% (variant-pool), and 42.6% (variant-tetramer). 75 anti-HCV negatives were scored negative by all the coating antigens evaluated.

### Anti-HVR1 antibody responses in mice immunized with individual HVR1-variants

100% seroconversion was observed for all the variants except variant-2. For variant-1, anti-HVR1 antibody titres were independent of the dose or addition of pNS3 (Figure 2a). However, after second dose, the antibody titres in 50 μg peptide + DNA group (p = 0.000) and 50 μg peptide + pNS3 group (p = 0.000) were significantly higher than 25 μg peptide + DNA group. A similar pattern was observed for variants-3 and 4 (Figure 2c, d). Antibody titres against variant-1 were significantly higher than variant-3 (p = 0.000) in the 50 μg peptide + DNA groups and 50 μg peptide + pNS3 groups (p = 0.001) after 2^nd ^dose. Anti-HVR1 titres were higher for variant-4 than variant-3 in 50 μg peptide + DNA group (p = 0.031) after 2^nd ^dose. No significant difference in the anti-HVR1 antibody titres was observed among different variant groups (p > 0.05) after 3^rd ^dose, except in variant-3 and variant-4, 25 μg peptide group (p = 0.009).

**Figure 1:**
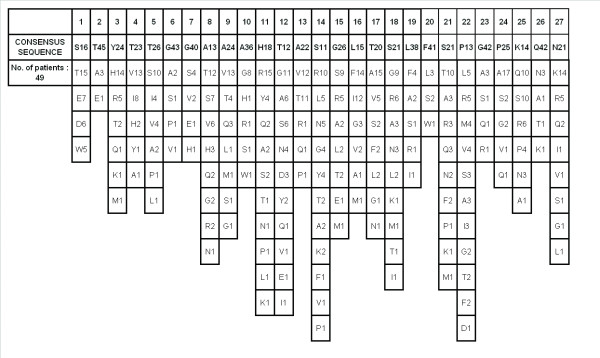
**HVR1 amino acid consensus profile obtained from 49 chronically infected hepatitis C patients**. On an average, 20 HVR1 clones were sequenced for each patient. The numbers against each amino acid indicate the number of samples in which that particular amino acid was present. Consensus sequence is given in bold.

**Figure 2:**
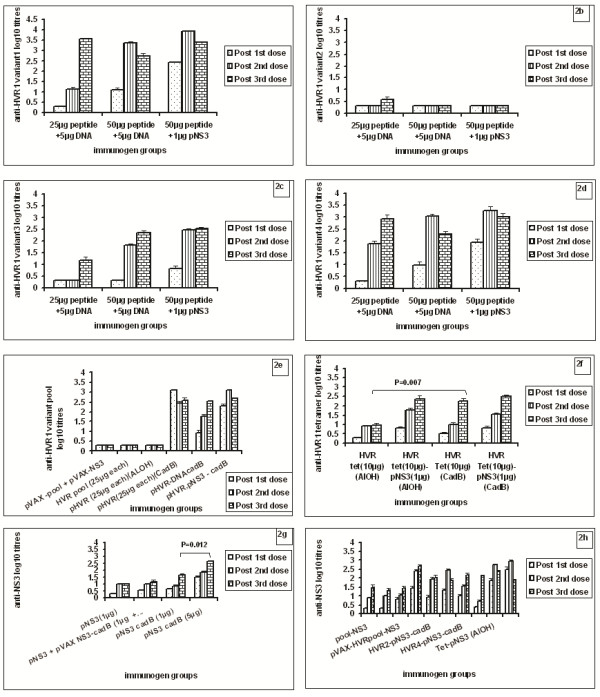
**Serum anti-HVR1 and anti-NS3 IgG titres (mean ± SE values) at 2 weeks post first, second and third dose in mice immunized with different antigen formulations**. Values are represented as mean log 10 titre ± SE. *p-Value < 0.05 (*t*-test). a to 2 d: Anti-HVR1 antibody titres in mice immunized with individual HVR1 variants 1-4 respectively. e: Anti-HVR1 antibody titres in mice immunized with HVR1 variant pool. f: Anti-HVR1 antibody titres in mice immunized with HVR1 variants tetramer. g, 2 h: Anti-NS3 antibody titres in mice immunized with different doses of recombinant pNS3, rpNS3 in combination with HVR1 component.

### Anti-HVR1 antibody responses in mice immunized with HVR1-variant-pool

In mice immunized with either pool-peptides + DNA, or pool-peptides + pNS3, 100% seroconversion was noted after 2^nd ^dose. No seroconversion was seen when variant-pool was given alone or with Al(OH)_3_. Interestingly, 100% seroconversion was recorded when variant-pool was given along with CadB. Immunization with DNA alone failed to produce antibodies. The anti-HVR1 antibody titres of pHVR1-pool-CadB, pVAX-pHVR-pool-CadB and pHVR1 pool-pNS3-CadB were comparable (p > 0.05). HVR-pool + pNS3 administered with CadB produced higher antibody titres than without adjuvant (p = 0.002) (Figure 2e). Addition of DNA or pNS3 along with peptides did not alter antibody titres (p > 0.05).

### Anti-HVR1 antibody responses in mice immunized with HVR1-variant-tetramer

100% seroconversion was recorded in all groups after second dose except when 10 μg tetramer was given with Al(OH)_3 _(75% seroconversion after 3 doses). Anti-HVR1-antibody titres were comparable for tetramer-CadB, tetramer-pNS3-CadB, tetramer-Al(OH)_3 _and tetramer-pNS3-Al(OH)_3 _groups (p > 0.05). Anti-HVR1-antibody titres were significantly higher for tetramer-CadB group than tetramer-Al(OH)_3 _group (p = 0.007). Antibody titres for tetramer-pNS3-Al(OH)_3 _and tetramer-pNS3-CadB groups were comparable after second dose (p > 0.05). The antibody titres for peptide-pool-pNS3-CadB and tetramer-pNS3-CadB were comparable after third dose (p > 0.05). (Figure 2f)

### Anti-NS3p antibody response in mice immunized with pNS3

Mice immunized with pNS3 without adjuvant served as controls. 100% seroconversion was recorded when 5 μg pNS3 was administered with CadB while pNS3-DNA-CadB and pNS3-1 μg-CadB yielded 87.5% seroconversion; DNA-CadB alone led to poor response (50%). pNS3 administered with individual HVR1-variants, HVR1-variant pool or tetramer either with CadB or Al(OH)_3 _led to 100% seroconversion. Without adjuvant, 66.6% seroconversion was noted when NS3 was given with HVR1-variant-pool or tetramer. There was no significant difference in anti-NS3 antibody titres when pNS3 was given with or without CadB. The anti-NS3 titres were significantly higher for pNS3-5 μg-CadB than pNS3-1 μg-CadB group after first (p = 0.012), second (p = 0.002) and third doses (p = 0.009) (Figure 2g). Anti-NS3 antibody titres were comparable for HVR1-variant-pNS3, HVR1-variant-pool-pNS3, and tetramer-pNS3 combinations (p > 0.05). The anti-NS3 titres for pVAX-NS3 group were comparable to that receiving pNS3 alone (p > 0.05) (Figure 2h).

### Antibody isotype profiling

#### Individual HVR1-variants

**Figure 3:**
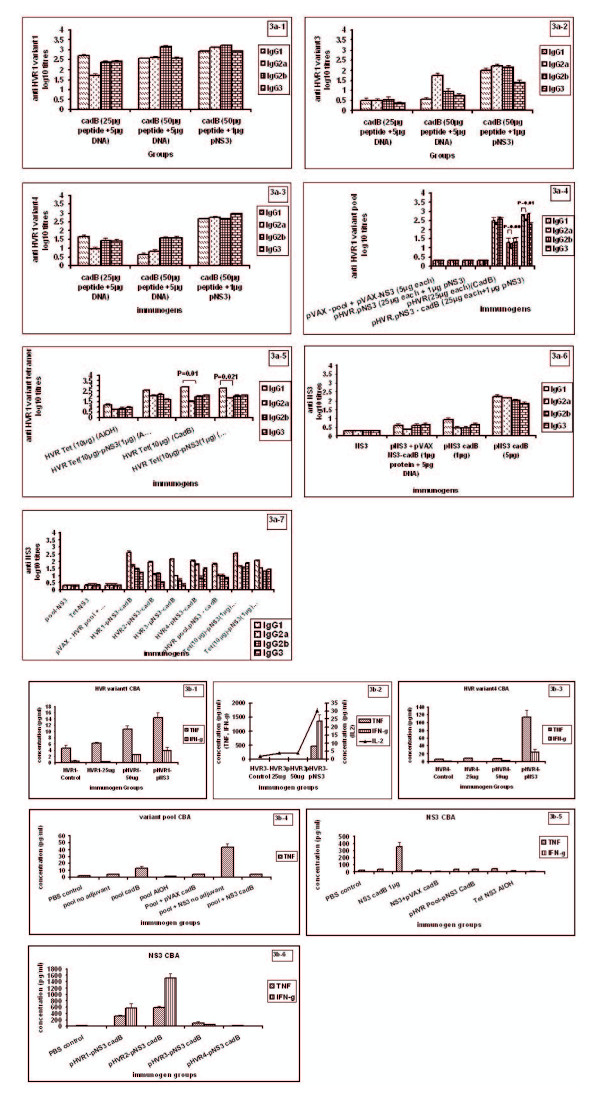
**a Serum anti-HVR1 and anti-NS3 IgG isotype titres at 2 weeks post-third dose in mice immunized with different antigen formulations at 0, 4 and 8 weeks**. Values are represented as mean log 10 titre ± SE. *p-Value < 0.05 (*t*-test). a-1 to 3a-3: Serum antibody isotyping for individual HVR1 variants (variants 1, 3, and 4 respectively). a-4: Serum antibody isotyping for HVR1 variant pool. a-5: Serum antibody isotyping for HVR1 variant tetramer. a-6, 3a-7: Serum antibody isotyping for NS3. **b: **Cytokine profile in spleenocytes after in vitro stimulation with either HVR1 peptides or recombinant pNS3 at 2 weeks post-third dose in mice immunized with different antigen formulations at 0, 4 and 8 weeks. Values are represented as mean (pg/ml) ± SE. *p-Value ≤ 0.05 (Mann-Whitney test). b-1 to 3b-3: cytokine profile after stimulation with individual HVR1 variants1, 3, 4. b-4: cytokine profile after stimulation with HVR1 variant pool. b-5, 3b-6: cytokine profile after stimulation with recombinant pNS3.

Anti-HVR1-IgG antibodies included all the four isotypes (IgG1, IgG2a, IgG2b and IgG3) suggesting involvement of both Th1 and Th2 pathways. In variant1-25 μg group, IgG1 titres were significantly higher than IgG2a titre (p = 0.011) indicating skewing towards Th2 response. However, with the increase in dose from 25 μg to 50 μg and also with the addition of pNS3, titres against IgG1 and IgG2a isotypes were comparable suggesting balanced Th1/Th2 response (p > 0.05) (Figure 3a-1). For variant-3, 50 μg peptide + DNA group showed an increase in IgG2a titre than IgG1 titre (p = 0.017) indicating Th1 type response. Titres for other groups of variant-3 were comparable (p > 0.05) (Figure 3a-2). When variant-4 was used as an antigen, for the 25 μg group, IgG1 was detected in higher titres than IgG2a (p = 0.047), indicative of Th2 response. IgG isotype titres were comparable for 50 μg peptide + DNA and 50 μg peptide + pNS3 groups (p > 0.05) suggestive of balanced Th1/Th2 response (Figure 3a-3).

### HVR1 variant pool

**Figure 4:**
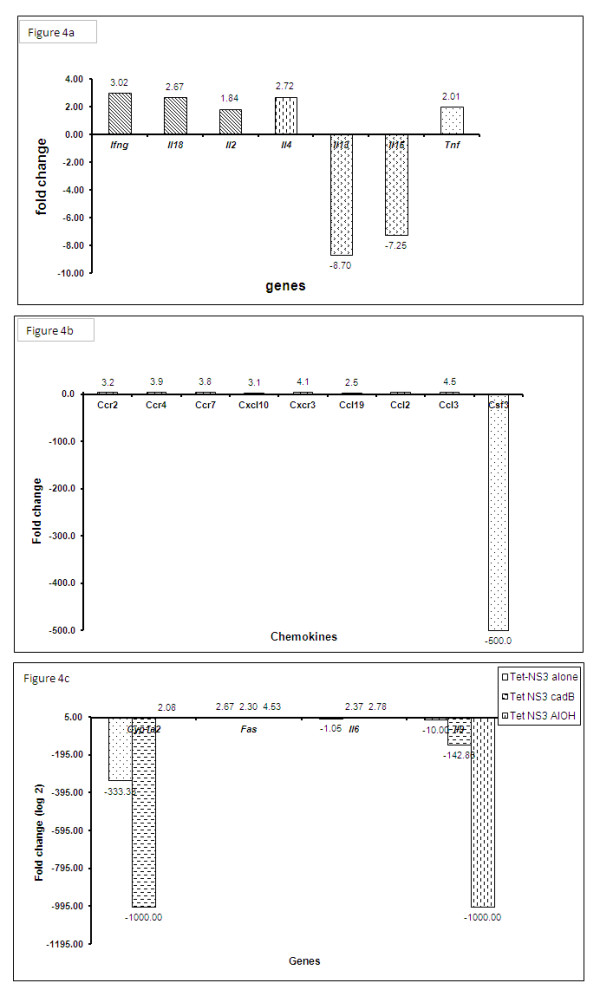
**a, b. Gene up regulation/down regulation (fold change) values for pool-NS3-cadB group when pool-NS3 without adjuvant was used as calibrator**. Th1: IFN-g, Il18, Il2. Th2: Il4, Il13, Il15. Pro-inflammatory: Tnf. Chemokines: Ccr2, Ccr4, Ccr7, Cxcl10, Cxcr3, Ccl19, Ccl2, Ccl3, Csf3. **c: **Gene up regulation/down regulation (fold change) values for tetramer-NS3-cadB, tetramer-NS3-Al(OH)_3_, tetramer-NS3 without adjuvant groups with PBS control as calibrator.

All the four isotypes IgG1, IgG2a, IgG2b and IgG3 were detected for the various groups. In HVR1-variant pool-CadB group, comparable IgG1 and IgG2a titres were obtained (p > 0.05) indicating a balanced immune response. In both HVR1-variant pool-DNA-CadB group (p = 0.007) and HVR1-variant pool-pNS3-CadB group (p = 0.010), IgG1 titres were significantly higher than IgG2a titres indicating skewing towards Th2 response (Figure 3a-4).

### HVR1 variant tetramer

**Figure 5:**
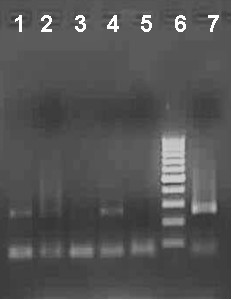
**Lane 1: mouse serum obtained after inoculation with HVR peptide variant pool (genotype 3a virus)**. Lane 2: Prebleed serum tested against genotype 3a virus. Lane 3: Negative control. Lane 4: mouse serum obtained after inoculation with HVR1 peptide variant pool (genotype 1b virus). Lane 5: Prebleed serum tested against genotype 1b virus. Lane 6: Molecular weight marker. Lane 7: Positive control 250 bp band corresponding to the diagnostic PCR positive control was detected.

All four isotypes (IgG1, IgG2a, IgG2b and IgG3) were detected indicating involvement of both Th1 and Th2 type immune response. In tetramer-CadB (p = 0.012) and tetramer-pNS3 CadB (p = 0.021) groups, IgG1 was detected in higher titres than IgG2a, while IgG1 and IgG2a titres were comparable when Al(OH)_3 _was used along with HVR1-tetramer (p > 0.05) (Figure 3a-5).

### Recombinant NS3p

Both cell mediated and humoral response was indicated as all the four isotypes were detected. When pNS3 was given along with HVR1-variants-1 or 3 or HVR1-variant-pool-CadB or tetramer-Al(OH)_3_, higher IgG1 titres were detected when compared to IgG2a titres (p = 0.027, 0.003, 0.007, 0.001 respectively). Balanced response was seen in variant-2-pNS3, variant-4-pNS3 and tetramer-pNS3-CadB groups with comparable IgG1 and IgG2a titres (p > 0.05). The pVAX-NS3-variant-pool-CadB group also showed balanced immune response with comparable IgG1 and IgG2a titres (p > 0.05) (Figure 3a-6, 3a-7).

### Cytokine bead array

For the assessment of the type of immune response (Th1/Th2/balanced) generated by the different formulations, secretion of specific antigen-stimulated cytokines, i.e., TNF (pro-inflammatory), IL-2/IL-4 (Th1) and IFN-g/IL-5 (Th2) was determined (Figure 3b). Overall, except for TNF and IFN-g, induction of other molecules was not recorded. For HVR1-variant groups, combination with NS3 did show secretion of IFN-g and TNF. Variants 1 and 4 produced higher levels of TNF than IFN-g. However, variant-3-pNS3 induced high levels of IFN-g (1348.8 ± 233.8 pg/ml) when compared to TNF (460.9 ± 25.6 pg/ml). IL2 was also detected (30.3 ± 1.9 pg/ml). Addition of corresponding DNA did not influence cytokine stimulation (Figure 3b). In accordance of absence of antibody response, no cytokines were detected for the variant-2 group. On the contrary, despite higher antibody titres, no cytokines were detected in the tetramer-group. Immunization with variant pool + NS3 in the presence/absence of the adjuvant was associated with the no secretion/secretion of TNF respectively. As far the response with respect to pNS3 is concerned, variant-1-NS3, Variant-2-NS3, pool-Ns3-CadB, pool-NS3/tetramer-NS3 without adjuvant and NS3 with/without adjuvant led to TNF response (Figures 3b). Upon stimulation with pNS3, IFN-g response was noted only with variant-1 (581.9 ± 130 pg/ml) and 2 (1515.5 ± 143.7 pg/ml).

### Taqman low density array (TLDA)

Upon comparing the genes of the TLDA mouse immune panel for control groups immunized with PBS, cadB and aluminium hydroxide with unimmunized control group, no significant up regulation or down regulation was noted. This indicated that the adjuvants alone were not responsible for regulation of the genes.

When pNS3 - HVR1 variant pool was used as a calibrator for pNS3 - HVR1 variant pool - cadB group, the following genes were up regulated in the liposome encapsulated group (fold changes). A difference >/= 2 folds was considered significant. Th1: IFN-g (3.0), Il18 (2.7), Th2: Il4 (2.7) Pro-inflammatory: TNF (2.0) Chemokines: Ccl19 (2.6), Ccl2 (5.0), Ccl3 (4.5), Ccr2 (3.2), Ccr4 (3.9), Ccr7 (3.8), Cxcl10 (3.1), Cxcr3 (4.1), and Others (Cd19, Cd28, Cd38, Cd4, Cd40, Cd40Ig, Cd8a, Col4a5, Csf1, Csf2,, Ece1, Edn1, Gzmb, H2-Eb1, Hmox1, Icos, Ifbkb, Il7, Il15, Prf1, Ptgs2, Selp, Smad3, Smad7, Socs1, Stat4, Stat6, Tbx21, Tnfsrf18. Nfkb2). Th1 gene Il2 though not significant, was seen to be marginally upregulated (1.8). Th2 genes Il13 (8.7), Il5 (7.3), and chemokine Csf3 (500), were down regulated (Figures 4a, b).

Overall, it was seen that the immune response was skewed towards Th1 type as suggested by the up regulation of IFN-g, Il18, and Il2 genes. This was further supported by the up regulation of pro-inflammatory TNF and chemokine genes (Ccl19, Ccl2, Ccl3, Ccr2, Ccr4, Ccr7, Cxcl10, Cxcr3). The Th2 genes Il13, Il5 were down regulated. The up regulation of Il4 suggested generation of Th2 immune response as well.

On comparing the groups HVR1 variant pool without adjuvant with HVR1 variant pool given along with cadB or aluminium hydroxide, there was no significant difference in the gene profile (data not shown). This further demonstrates the combined action of NS3 and cadB that led to the immune response pattern in the pool-NS3-cadB group as described above.

When Tetramer-NS3 without adjuvant, tetramer-NS3-cadB and tetramer-NS3-aluminium hydroxide groups were calibrated against PBS, the fas gene was up regulated in all three groups. In tetramer-NS3 without adjuvant group, cyp1a (333.3 fold) gene and Il9 (10 fold) were down regulated. In tetramer-pNS3-cadB group cyp1a (1000 fold) and Il9 (142.9 fold) genes were down regulated and fas (2.3 fold) and Il6 (2.4 fold) genes were up regulated (Figure 4c). No significant difference was recorded for other genes. Il9 was consistently down regulated in all three groups (tetramer-NS3: 10 fold, tetramer-NS3-cadB: 142.9 fold, tetramer-NS3-Al(OH)_3_: 1000 fold). Il6 was not significantly altered in the tetramer-NS3 without adjuvant group. However, Il6 expression was marginally up regulated upon addition of either cadB (2.4 fold) or Al(OH)_3 _(2.8 fold). Based on the expression pattern of these genes, Th1/Th2 immune response pattern could not be conclusively determined for tetramer-NS3-cadB, tetramer-NS3- Al(OH)_3 _or tetramer-NS3 without adjuvant as immunogen groups.

### Immune capture-RT PCR

Anti-variant-pool antibodies could bind both genotype 3a and 1b viruses as detected by the immune-capture-RT PCR (Figure 5).

## Discussion

Though the exact mechanisms of protection against HCV infection remain unclear, association of both humoral and cellular immune responses have encouraged evaluation of different approaches for vaccine development. Over the last decade, numerous HCV vaccine approaches have been assessed in mice and primates. So far, majority of the recombinant candidate vaccines have been predominantly generated from genotype 1 virus [31,32]. Many of these vaccine attempts including those reaching clinical trials produced cross-genotypic immune responses [29,31,33]. Still, the fact remains that the vaccine-genotype specific antibodies are mainly produced.

The predominance of genotype 3 in India (> 70%) [9] led us to attempt to develop a genotype 3 based vaccine. The two components of our candidate vaccine were the hyper variable region 1 (HVR1) and non-structural protein-3 (NS3).

The E2 glycoprotein harbours HVR1, a highly diverse region of the HCV genome. Taking into consideration the high mutation rate of the HVR1 region due to host as well as viral components [34-36], we determined consensus HVR1 sequence based on the genotype-3 HCV sequences from different parts of India. For this, we used samples from all available subtypes of genotype 3 in the frequency at which they were detected in the population (3a (27, 55.1%), 3b (8, 16.3%), 3e (2, 4.1%), 3f (2, 4.1%), 3 g (8, 16.3%), and 3i (2, 4.1%)).

The next issue to be addressed was the presence and distribution of HCV quasispecies in the samples studied. A shift over time of the minor quasispecies to the most predominant one has been reported earlier [37]. Understanding the degree of variation and proportion of the different sequences was indeed necessary. For this, the quasispecies pattern was determined for all the 49 patients by sequencing 20 HVR1 clones obtained from every patient. The pattern varied from 2 to 14 quasispecies demonstrating degree of sequence variability in different patients and need to consider these variations for designing appropriate 27 amino acid long HVR1 peptides.

In the HVR1 consensus profile generated during this study, amino acid positions 2, 6, 7, 10, 19, 20, 23, and 26 were found to be more conserved than the rest (91.8% at position 2 to 73% at position 10% conservation). Amino acid positions 11, 12, 14, 22 and 25 were found to be most variable (77.6% at position 14 to 63.3% at position 11). Importantly, except for position 10, all the other relatively more conserved amino acids were also present in the consensus sequences reported earlier [33,38]. The preference of amino acids in a given position of HVR1 provides information as to the combinations of permissible mutations in any position of HVR1 [33,38]. This significant observation encouraged us to proceed further and design possible variants of the HVR1 sequence representing genotype 3 and also expect cross-reactivity with the other circulating genotypes.

A total of 4 variants were thought to cover the range of sequence variability. Before assessing the immunogenecity of the 4 peptide variants in mice, we examined immunoreactivity with antibodies from 115 anti-HCV positive patients' sera infected with different genotypes (genoytpes 3, 1, 4 and 6). ELISAs were performed using the peptides individually, as a pool or as a single tetramer where the individual peptide sequences were used in tandem interspersed with 4 glycine residues. Overall, all the variants efficiently detected genotype 3 and a substantial proportion of genotype 1 infected sera (Table 1). Use of variant pool did not improve ELISA performance, and in fact, resulted in lower reactivity than the individual variants 1, 3 and 4 probably because of the competitive inhibition. The tetramer exhibited lowest reactivity (42.6%) with the anti-HCV positive human serum samples when compared with the peptide pool (72.2%). ELISA results showed that the HVR1 peptides used exhibited a high degree of cross-genotypic reactivity and could be taken forward for the immunogenicity studies. A substantial proportion (26/40) of anti-HCV positives (genotypes 1, 2, 3 and 4) were earlier shown to be non-reactive with a peptide polytope comprising of variants of 11 N-terminal amino acids of HVR1 were used as coating antigens [29]. Our results encouraged us to evaluate the HVR1 consensus and its variants as candidate vaccine. Murine antibody response to HVR1 region was assessed for each variant individually, pool of all the 4 variants or as a tetramer. The variant-2, though immunoreactive, was not immunogenic in mice. This variant differed from the consensus sequence by 5 amino acids (positions 3, 8, 9, 10, 11), while the variants 3 and 4 that differed from the consensus by 3 (positions 3, 8, 9) and 1 (position 9) amino acids respectively were immunogenic. Probably, substitutions at four consecutive positions or substitutions at positions 10 and 11 rendered variant-2 non-immunogenic. Antibody responses to individual variants 1, 3 and 4 were comparable to the HVR1 variant pool and could be detected by all the variants individually, indicating cross-reactivity in ELISA. The results are in concordance with Mihailova et al [39], where a combination of bacterially expressed HVR1 tetramer, core and NS3 proteins were used as immunogens and high anti-HVR1 tetramer antibody titres and vigorous lymphoproliferative responses were obtained. Similar results were obtained when HVR1 N-terminal polytope [29], and HVR1 DNA [40] were used as immunogens.

An NS3 specific CD4+ T cell immune response is much stronger and more frequently found in patients who resolve acute hepatitis than in patients who develop chronic infection and this response may be necessary for virus clearance [41]. Importantly, several studies have demonstrated the efficacy of the NS3 region as a vaccine candidate either singly or in combination with other HCV proteins [42-46]. In our study 100% antibody seroconversion for anti-NS3 antibodies was seen after second dose. Addition of individual variants, variant pool or tetramer in Cad-B (liposome) yielded optimum and comparable antibody titres in mice. The enhanced anti-NS3 response by the addition of HVR1 is of particular advantage as we intended to use combination of HVR1 and NS3 as vaccine candidates.

As against our earlier observations of enhanced immune response following encapsulation of proteins and corresponding DNAs of hepatitis E and B viruses [25], no advantage was noted with both HVR1 and NS3. Thus, the phenomenon of enhancement of immunogenicity by DNA seems to be immunogen-dependent.

Adjuvanted pNS3 did produce significantly higher antibody titres. The effect with reference to adjuvant could be detected in NS3-HVR1 variant tetramer. 100% seroconversion was noted after third dose for tetramer-pNS3 given either with cadB or aluminium hydroxide, whereas 66.6% seroconversion was observed when NS3 was given with HVR1 variant pool or tetramer without any adjuvant.

On account of the inherent problems of the immunogenecity of peptides and recombinant proteins, use of adjuvants becomes necessary. In view of the crucial role played by various adjuvants in determining immunogenicity,[18,19], we decided to compare, liposomes (Cad-B) with Al(OH)_3_, the time-tested adjuvant. In our lab, liposome was successfully used in the development of recombinant hepatitis E vaccine as judged by experiments in mice and rhesus monkeys [25,47] as well as for a recombinant combination vaccine for hepatitis E and B [25]. Liposome encapsulation did enhance immunogenicity of the HCV antigens [42,48,49]. Though we were using 27-amino acids peptides as immunogens, formulations with Cad-B (HVR1 pool alone, HVR1-pool-NS3 and HVR1-pool + corresponding DNAs) yielded high antibody titres as compared to low/no antibodies with Al(OH)_3_. Further, even though the tetramer induced high antibody titres with different formulations, the lower efficiency (42.6%, Table 1) in detecting anti-HCV antibodies in human serum samples argues against its suitability as a vaccine candidate. Thus, liposome encapsulated HVR1 peptides emerged as suitable vaccine candidate.

As our aim was to use HVR1 and NS3 for reasons described earlier, it was important to assess the effect of adjuvants, the individual components and combination of these immunogens on the antibody response. As far as the antibody titres against NS3 component is concerned, addition of individual variants, variant pool or tetramer in Cad-B yielded optimum titres (Figure 2). Addition of pNS3 to the peptides did not alter anti-HVR1 antibody titres significantly (p > 0.05) as compared to the addition of corresponding DNA (Figure 2). This data strongly suggests utility of HVR1-NS3 combination.

Many reports of vaccine development for hepatitis C had used ELISPOT, LPA, intracellular staining and chromium release assays to determine the cell mediated responses against candidate vaccines [29,43,50]. In our experiments, we used cytokine bead array to determine the Th1/Th2 response pattern. We also substantiated this data by serum antibody isotype pattern, an indicator of the type of immune response obtained. Earlier studies reported detection of IFN-gamma (Th1 response) and IL4 (Th2 response) using ELISPOT for NS3 and other non-structural proteins [51,52]. However, we did not observe strong T-cell mediated immune response as judged by the induction of cytokines by antigen-stimulated spleenocytes using CBA. In the cytokine bead array analysis, activation of both Th1 and Th2 cytokines was seen though at very low concentrations. This was probably because mice spleens were harvested at a later stage (15 days after 3^rd ^dose). The possibility of higher elevation in these cytokines at early time points cannot be ruled out. Incidentally TNF-alpha the pro-inflammatory cytokine was detected at higher concentrations (to a maximum of 583.68 ± 51.34 pg/ml).

Isotyping analysis documented involvement of both Th1 and Th2 type immune responses with NS3 and Th2 with HVR1 component. Variant-1 and 4 with 1 amino acid substitution behaved similarly i.e., Th2 response with 25 μg variant + corresponding DNA switching to balanced Th1/Th2 isotype pattern after the increase of peptide to 50 μg or addition of 1 μg pNS3. The effect of Al(OH)_3 _and Cad-B adjuvants on isotype pattern could be studied for variant-pool and tetramer. Al(OH)_3_-variant-pool group remained antibody negative while addition of CadB led to balanced response. With tetramer, Al(OH)_3 _gave balanced response while Th2 response was observed with CadB adjuvant. It has been shown earlier that the composition of cationic liposomes resulted in a shift from Th1 to Th2 anti-NS3 antibody response. NS3 DNA given along with DDAB/DOEPC a highly charged cationic liposome combination was seen to induce Th2 response in comparison to DDAB/EPC liposome-NS3 DNA formulation which induced Th1 response. It was further hypothesized by Jiao et al [48] that plasmids adsorbed on muscle cells induced Th2 type of immune response while those captured directly by APCs would induce Th1 type of immune response. Thus the type of response was influenced by the adjuvant used and the site of injection [48].

We further checked the gene expression profile for mice immunized with various antigen formulations either with or without adjuvants using Taqman Low Density Array (TLDA) in order to confirm the results obtained in isotyping analysis.

The adjuvants, when given alone without antigens, did not show significant change in gene profiling when compared with PBS control, thereby indicating that the immune response of the antigen was enhanced/modified because of the adjuvanted antigens. The adjuvants (Al(OH)_3 _/CadB) by themselves did not cause alteration in gene regulation.

Both Th1 and Th2 genes were up regulated indicating a balanced immune response and its enhancement upon liposome encapsulation of HVR1 pool-NS3-cadB group. This conclusion was further supported by serum antibody isotypes. The absence of IL5 rise in spleen was accompanied by the down regulation of the gene.

On comparing HVR1 variant pool without adjuvant and HVR1 variant pool given either with CadB or aluminium hydroxide, no significant difference in the gene profile was recorded. Thus the observed gene profile could be attributed to the additive effect of NS3, CadB and HVR1 pool. A similar phenomenon was not observed with the tetramer-NS3 combinations, indicating that individual peptides rather than the tetramer could generate better cytokine response, despite similar antibody titres produced by anti-HVR1 pool and anti-HVR1 tetramer. This further strengthens our conclusion that HVR1 variant pool, and not HVR1 tetramer should be used for further studies. Overall, it was observed that the HVR1 pool-NS3 combination encapsulated in CadB was the most promising vaccine candidate to be studied in depth.

In the absence of an easy, efficient and convenient in-vitro/in-vivo neutralization test system, we performed the immune capture RT-PCR for the demonstration of binding of the virus to the antibodies generated against the HVR1. It is indeed important to note that these antibodies were able to bind viruses of genotypes 3a and 1b. Whether this binding is indicative of neutralization cannot be ascertained. Similar results were reported with HVR1 based polytope vaccine candidate [29].

In conclusion, the combination of peptides of HVR1 consensus sequence and its variants along with pNS3 encapsulated in liposomes could generate both cellular and humoral immune responses in mice deserving further evaluation in a suitable cell culture system/non-human primate model.
